# Factors associated with apparent efficiency of absorption of immunoglobulin G in dairy and beef-cross calves: A cohort analysis

**DOI:** 10.3168/jdsc.2026-1083

**Published:** 2026-10-05

**Authors:** R. Davies, D.L. Renaud, A.I. Macrae

**Affiliations:** 1 Veterinary Technical Consulting Ltd., Haverfordwest, Wales, SA61 2BP, United Kingdom; 2 Department of Population Medicine, University of Guelph, Guelph, ON N1G 2W1, Canada; 3 Dairy Herd Health and Productivity Service, Royal (Dick) School of Veterinary Studies and the Roslin Institute, The University of Edinburgh, Easter Bush Campus, Roslin, Midlothian, EH25 9RG, Scotland, United Kingdom

## Abstract

Ensuring successful transfer of passive immunity remains a major challenge in pasture-based block-calving systems. This cohort study aimed to evaluate factors associated with apparent efficiency of absorption (AEA) of IgG in 213 dairy and beef-cross calves born on a spring block-calving pasture-based dairy herd in West Wales from February to May 2024. All calves were removed from the dam at birth and fed colostrum replacer and pooled colostrum and transition milk within the first 24 h of life. Serum IgG concentrations at 24 to 36 h and the IgG concentrations of the colostrum replacer and each pooled colostrum and transition milk batch were measured by radial immunodiffusion and used to calculate AEA. A multivariable linear regression model was then used to evaluate associations between AEA and potential explanatory calf- and management-level variables. Calves born during the morning (+5.49 percentage points; 95% CI: 1.56 to 9.41) and afternoon or evening (+5.35 percentage points; 95% CI: 1.46 to 9.24) had a higher AEA than those born overnight. Greater total IgG intake during the first 24 h was associated with lower AEA (−0.10 percentage point change in AEA for every 1 g of IgG fed, 95% CI: −0.14 to −0.05). There was no evidence of an association of AEA with breed, sex, birth weight, or calving ease. Together, these results suggest that calving management and staffing patterns may influence AEA in pasture-based block-calving dairy herds.

## Graphical Abstract

**Figure:**
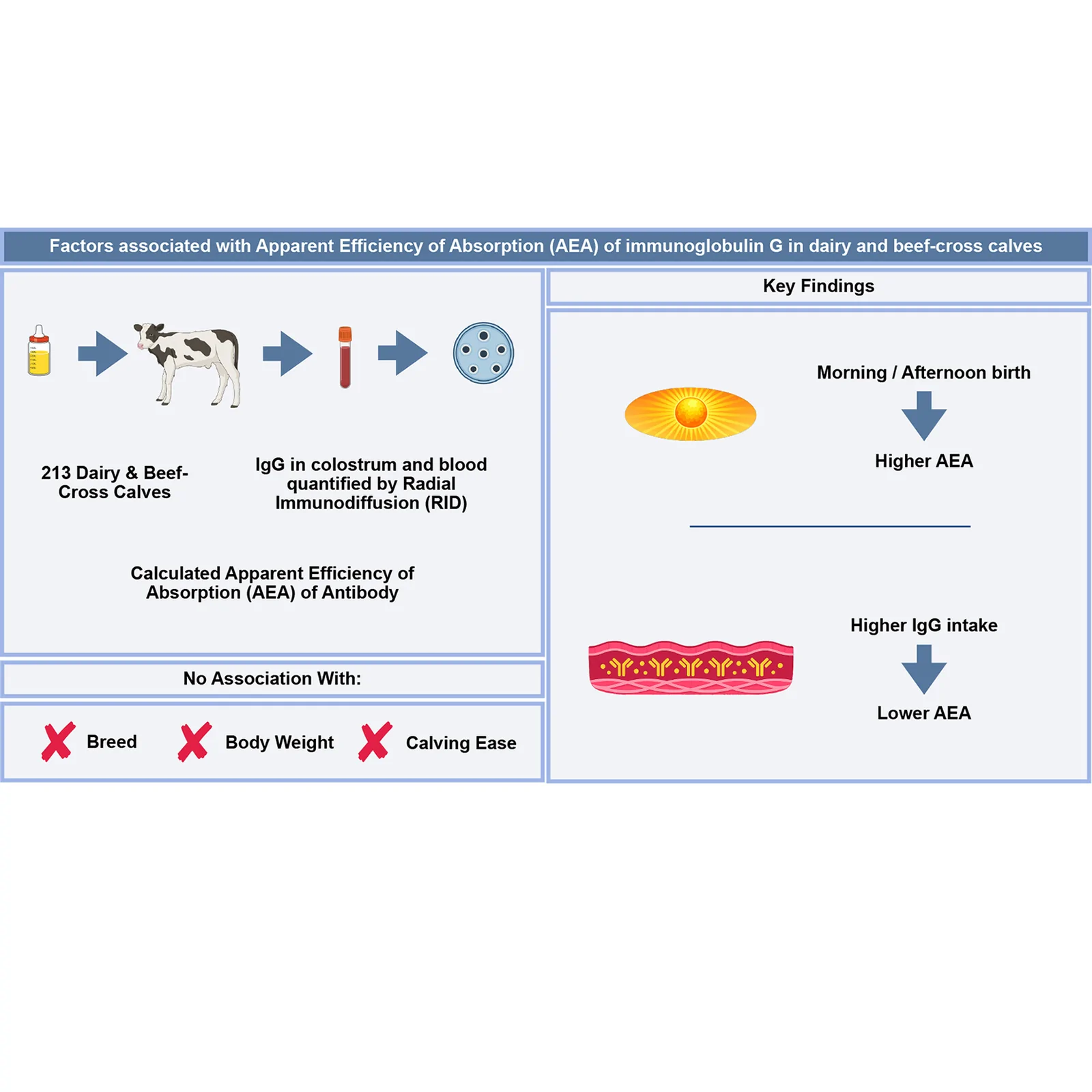

Successful transfer of passive immunity is essential for calf health and survival, as neonatal calves rely on the absorption of IgG from colostrum to establish early immune protection (Godden et al., 2019). Despite improvements in colostrum management, failure of transfer of passive immunity (**FTPI**; serum IgG <10 g/L) remains common in dairy production systems. A recent meta-analysis in Australasia in pasture-based dairy systems reported that 33% of dairy calves experienced FTPI, with substantial variation between farms (Van et al., 2023). Comparable estimates from more intensive indoor systems report lower prevalence, including 15.6% in the United States (Urie et al., 2018) and 24% in Canada (Renaud et al., 2020). In the United Kingdom, where both systems exist, a recent cross-sectional study reported a FTPI prevalence of 26% in predominantly year-round calving herds (Lindley et al., 2025).

In pasture-based block-calving dairy production systems, colostrum management practices likely contribute to this variability. In some farms, calves may obtain colostrum directly from the dam through suckling before collection and feeding interventions occur (Mason et al., 2022), although colostrum intake under these conditions is often variable in timing, quantity, and quality. Even when calves are subsequently fed colostrum in rearing facilities, delays to first feeding and suboptimal colostrum quality remain common (Denholm et al., 2017). As a result, variability in passive immunity in this system is driven by differences in suckling behavior as well as colostrum delivery, particularly timing and IgG intake (Mason et al., 2022).

A key determinant of passive immunity is the apparent efficiency of absorption (**AEA**) of IgG, which is a derived measure calculated from serum IgG, estimated blood volume based on birth weight, and IgG intake. Substantial variation in AEA has been reported in indoor dairy systems. For example, Halleran et al. (2017) reported AEA values ranged from 7.7% to 59.9% with a mean of 28.1% ± 9.5% among Holstein calves despite standardized feeding protocols. Several factors have been identified that influence AEA, including time at first feeding (Fischer et al., 2018), IgG concentration and volume of colostrum fed (Frederick et al., 2025), and bacterial contamination of colostrum (Gelsinger et al., 2015). Additional factors such as sex, breed, dystocia, and environmental conditions may also affect IgG absorption (Quigley et al., 1995).

Despite the importance of AEA in determining passive immunity, limited information exists on IgG absorption efficiency in calves born into pasture-based block-calving dairy systems. The objective of this study was to determine factors associated with AEA of IgG in dairy and beef-cross dairy calves born into a pasture-based block-calving dairy herd.

The study was conducted as part of a previously published study evaluating the effect of passive immunity and transition milk quality in a pasture-based spring block-calving herd (Davies et al., 2025). A total of 251 calves were initially enrolled at a single commercial dairy herd in Pembrokeshire, West Wales from February to May of 2024. Calves were removed from their dams as soon as they were identified, and the date and time of birth, twin status, sex, breed, and birth weight (Dairy Calf Tape, The Coburn Company Inc., Whitewater, WI) were recorded. Calving ease was scored on a scale of 1 to 4, with 1, 2, 3, and 4 corresponding to unassisted, easy pull, hard pull, and surgery, respectively (CanWest Dairy Herd Improvement System, Ontario, Canada).

Calves were monitored using an overhead video camera in the maternity pen (Farmer's Eye, Dundee, Scotland, UK), and those observed to suckle from their dam were excluded from the analysis (n = 35). An additional 3 calves were excluded due to incomplete monitoring after cows were moved outdoors to calve at the end of the calving period. These calves were excluded because total IgG intake could not be reliably quantified. Compared with included calves, excluded calves had substantially higher calculated AEA values, consistent with additional unmeasured IgG intake from suckling and supporting their exclusion from the analytical AEA dataset. Excluded calves were represented across months of birth, time of birth categories, breeds, birthweights, and calving scores, with no evidence that exclusions were concentrated within a particular subgroup. Although excluded calves were broadly similar to included calves with respect to the measured characteristics, the potential for selection bias cannot be completely excluded. The final analytical dataset comprised 213 calves: 119 British Friesian dairy calves (100 females and 19 males), 77 Wagyu × British Friesian F_1_ calves (27 females and 50 males), and 17 Hereford × British Friesian calves (4 females and 13 males), for a total of 131 females and 82 males.

For their first feed, each calf received 1 to 2 sachets (700 g) of whole bovine spray-dried colostrum (Calf's Choice Total 100, Saskatoon Colostrum Company Ltd., Saskatoon, SK, Canada), each containing at least 100 g IgG. Each sachet was added to 1.25 L of water at 45°C from an electrical immersion heater to make a 2-L solution and administered via esophageal feeding tube. The number of sachets administered to each calf was recorded but was not assigned according to a formal study protocol. The number of sachets administered reflected differences in routine practice among farm staff, with some staff members routinely administering one sachet and others routinely administering 2 sachets based on personal preference and previous experience. The specific reason for administering 2 sachets to an individual calf was not recorded, and the decision was not based on a predefined assessment of calf vigor, birth weight, disease risk, or other calf-level characteristics. When 2 sachets were administered, both sachets were reconstituted together into a single 4-L solution and administered via esophageal feeding tube. Subsequent feedings within the first 24 h of life consisted of a 2.5-L mix of pooled colostrum and transition milk (milkings 2–6 after calving; Godden et al., 2019) and were fed via teat feeder. Calves received 2 to 3 feedings of this mixture in addition to their first feed of colostrum replacer (**CR**) within the first 24 h of life. The number of subsequent feeds reflected timing of birth and feeding schedules rather than a randomized allocation.

Pooled colostrum and transition milk were harvested twice daily and heat-treated at 60°C for 60 min using a 270-L commercial pasteurizer (MilkTaxi, Holm & Laue, Westerrönfeld, Germany). A new pool was prepared for each feeding from colostrum and transition milk collected at the preceding milking. Following feeding, the pasteurizer was completely emptied and cleaned before preparation of the subsequent pool ∼12 h later. A postpasteurization sample from each pool was collected in a 30-mL sterile universal leakproof plastic container (International Scientific Supplies Ltd., Bradford, England), clearly labeled, immediately frozen at −20°C, and stored frozen until shipment to the Dairy Herd Health and Productivity Service (**DHHPS**; University of Edinburgh, Scotland) for determination of IgG concentration using radial immunodiffusion (**RID**). Calves receiving each pool were identified by ear tag number. The measured IgG concentration of each pool was then used to calculate total IgG intake during the first 24 h of life. Because calves received multiple pooled colostrum and transition milk feedings during this period, individual calves could receive milk from more than one sequential pool. Colostrum replacer was also submitted to the DHHPS for RID analysis.

Blood samples for determination of serum IgG concentration were collected from all calves between 24 to 36 h of age and at least 6 h after the final colostrum and transition milk feed. Samples were collected using a 20-gauge 1-inch (2.54-cm) needle into a 5-mL serum activator clot evacuated tube (BD Vacutainer, VetWay, York, England), and barcode labels were used to record the calf ID. Within 1 h of collection, samples were centrifuged at 1,300 × *g* for 5 min at 20°C. Serum was then removed using a micropipette and transferred to 2 Eppendorf tubes (DNA LoBind tubes, Camlab Ltd., Cambridge, England) with freezer-proof labels (Tough-Tags, Thistle Scientific, Glasgow, Scotland). Samples were then frozen at −20°C and transported frozen in batches to the DHHPS laboratory at the University of Edinburgh (Edinburgh, Scotland) for RID analysis.

The DHHPS laboratory at the University of Edinburgh analyzed the IgG content of the calf serum, CR, and pooled colostrum and transition milk mix by RID, as described by Chelack et al. (1993), and more recently by Shivley et al. (2018). In brief, 4-µL samples of serum and pooled colostrum and transition milk (diluted using phosphate-buffered saline as appropriate), along with controls, were deposited into separate wells of a plate agarose gel containing a bovine IgG-specific antiserum, manufactured by Saskatoon Colostrum Company Ltd. All samples were analyzed in duplicate. Following diffusion, a precipitating ring was formed after 24 h, with a diameter proportional to the IgG concentration. The ring's diameter was then measured using a scale loupe and converted using a logarithmic formula to determine the IgG concentration of the sample.

For each calf, total IgG intake was calculated by summing the IgG mass provided in the initial CR feeding and all subsequent pooled colostrum and transition milk feedings, with IgG concentrations determined separately for each batch of pooled colostrum and transition milk by radial immunodiffusion.

The AEA of IgG was calculated for each calf according to Quigley and Drewry (1998), Quigley et al. (2002), and Saldana et al. (2019), as follows:

$$AEA\;\left(\%\right)\;=\;\frac{\left\{\left[0.09\times BW\;\left(kg\right)\right]\times serum\;IgG\;\left(\frac{g}{L}\right)\right\}}{total\;IgG\;consumed\;in\;first24\;h\;\left(g\right)}\times 100,$$

where 0.09 × BW (kg) estimates plasma volume (in L) for neonatal calves, serum IgG (g/L) was measured at 24 to 36 h of age, and total IgG consumed (g) is the sum of IgG from the first replacer feed plus the IgG from each subsequent pooled colostrum and transition milk feed.

All statistical analyses were conducted in Stata 18 (StataCorp LP, College Station, TX). Data were imported from Microsoft Excel into Stata 18 and checked for completeness. Descriptive statistics were generated on all explanatory variables in the dataset. Linearity between continuous variables and AEA was assessed using locally weighted scatterplot smoothing curves. Variables that did not meet the assumption of linearity were categorized into quartiles. A linear regression model was then used to evaluate associations between AEA and potential explanatory variables. In univariable analysis, time of birth (morning [0400–1159 h], afternoon or evening [1200–1959 h], and overnight [2000–0359 h]), month of birth, breed, sex, total IgG consumed in the first 24 h, calving ease score, and birth weight were examined. Explanatory variables were chosen based on biological relevance and availability from the dataset. Twin status was not evaluated as an explanatory variable because only 2 sets of twins (4 calves) were represented in the analytical cohort.

Variables with *P* < 0.20 in the univariable analysis were offered to the multivariable model; breed was also included due to its biological relevance. Model diagnostics included assessment of residual normality, homoscedasticity, leverage, Cook's distance, studentized residuals, and DFITS. Potentially influential observations were individually reviewed, and sensitivity analyses were performed where appropriate. The model failed to meet the assumption of homoscedasticity, and no transformation of the outcome or predictor variables corrected the violations. To address this, robust standard errors were used to adjust confidence intervals and *P*-values for heteroscedasticity.

Most calves were born in February (75 calves, 35.2%), March (69 calves, 32.4%), and April (64 calves, 30.1%), with only 2.4% (5 calves) born in May. The mean (±SD) body weight was 39.3 ± 4.4 kg, corresponding to an estimated plasma volume of 3.5 ± 0.4 L. Median serum IgG concentration at 24 to 36 h of age was 12.9 g/L (interquartile range [**IQR**]: 8.9 to 14.7), with 77 calves having FTPI (defined as serum IgG <10 g/L), 104 in the “fair” category (serum IgG 10–17.9 g/L), 18 in the “good” category (18–24.9 g/L), and 14 in the “excellent” category (serum IgG ≥25 g/L), following Lombard et al. (2020). The total amount of IgG fed in the first 24 h was 184.8 ± 33.1 g. At the initial colostrum feeding, most calves (197/213; 92.5%) received a single CR sachet providing 96.3 g IgG, whereas 16 calves (7.5%) received 2 sachets (192.6 g IgG). Consequently, the median (IQR) IgG intake at the first feeding was 96.3 g (96.3 to 96.3 g). Subsequent feedings provided an additional 81.2 ± 28.5 g IgG. The calculated AEA across all calves was 24.9% ± 11.6%.

In the final multivariable model (Table 1), calves born in the morning (+5.49 percentage points; 95% CI: 1.56 to 9.41; *P* = 0.007) and afternoon or evening (+5.35 percentage points; 95% CI: 1.46 to 9.24; *P* = 0.007) had higher AEA compared with calves born overnight (Figure 1). Additionally, greater IgG intake in the first 24 h was associated with lower AEA, with a −0.10-percentage point change in AEA for every 1-g increase in IgG fed (95% CI: −0.14 to −0.06; *P* < 0.001; Figure 2). In an exploratory comparison, calves born in May had higher AEA than calves born in February (+17.05 percentage points; 95% CI: 6.97 to 27.14; *P* = 0.001). However, this estimate was based on only 5 May-born calves and should therefore be interpreted cautiously. Breed, sex, birth weight, and calving score were not significantly associated with AEA.

**Table 1:** Final multivariable linear regression model evaluating associations between apparent efficiency of absorption (AEA) of IgG and calf- and management-level factors, using robust standard errors (n = 213)

Variable	Category	Calves (n)	β coefficient (95% CI)	*P*-value
Month	February	75	Referent	
	March	69	−0.12 (−3.81, 3.57)	0.95
	April	64	1.95 (−2.10, 5.99)	0.34
	May	5	17.05 (6.97, 27.14)	0.001
Total IgG intake during first 24 h (g)	Continuous	213	−0.10 (−0.14, −0.05)	<0.001
Time of birth	Overnight	49	Referent	
	Morning	79	5.49 (1.56, 9.41)	0.006
	Afternoon or evening	85	5.35 (1.46, 9.24)	0.007
Breed	British Friesian	119	Referent	
	Wagyu	77	−2.09 (−5.33, 1.15)	0.21
	Hereford	17	−4.76 (−10.69, 1.18)	0.12

**Figure 1:**
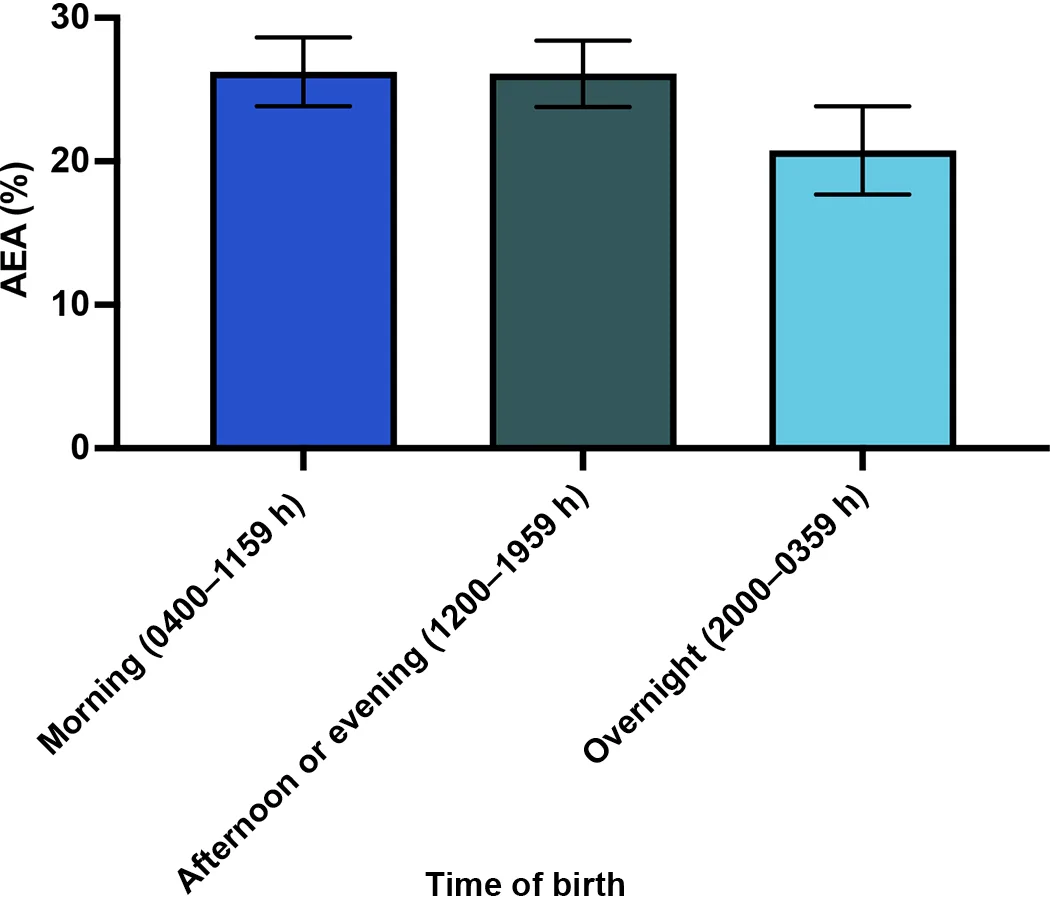
Model-predicted apparent efficiency of absorption (AEA) of IgG according to time of birth category (morning [n = 79], afternoon/evening [n = 85], and overnight [n = 49]). Values represent adjusted predictions from the final multivariable linear regression model, with 95% CI.

**Figure 2:**
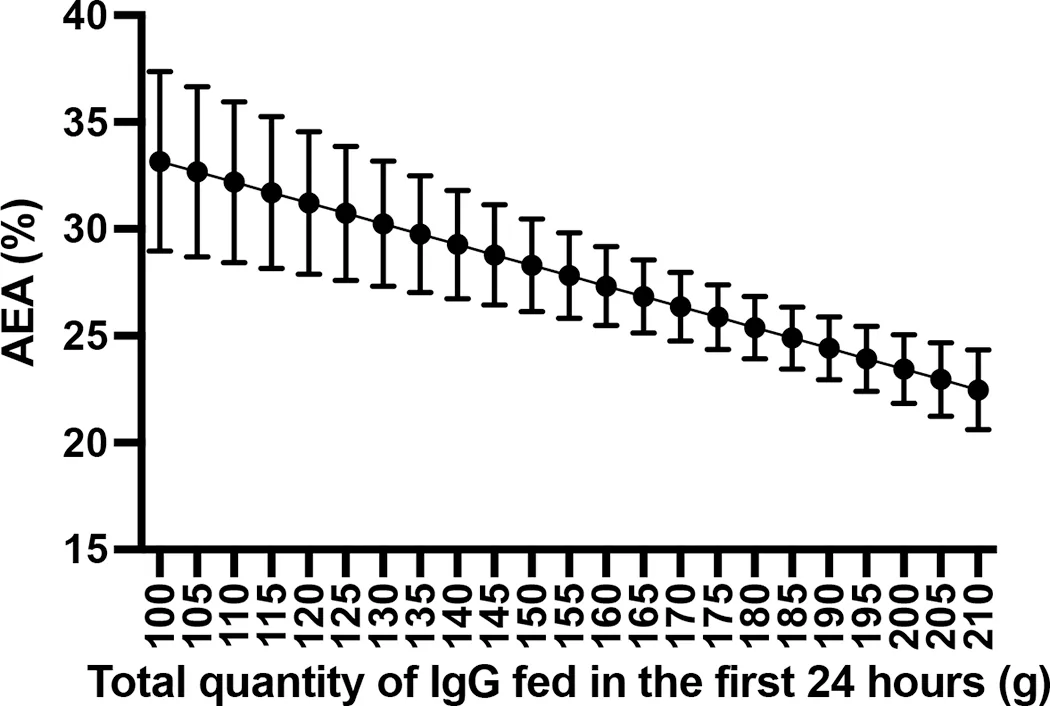
Model-predicted apparent efficiency of absorption (AEA) of IgG according to total IgG intake during the first 24 h of life. Values represent adjusted predictions from the final multivariable linear regression model, with 95% CI.

These findings suggest that AEA in pasture-based block-calving herds may be largely shaped by calving supervision and colostrum management practices, rather than inherent differences among calves. Higher total IgG intake during the first 24 h was associated with a lower AEA. However, total IgG intake forms the denominator of the AEA calculation and is therefore structurally related to the outcome. Consequently, the observed negative association may arise, at least partly, through mathematical coupling and cannot be interpreted as direct evidence of saturation of intestinal IgG transport. Despite this, biological saturation or diminishing proportional uptake at higher IgG intakes remain one possible explanation, consistent with previous studies (Quigley et al., 2002; Lopez et al., 2022; Mitman et al., 2025; Frederick et al., 2025), but the present study cannot distinguish this mechanism from the mathematical properties of the calculated outcome. Immunoglobulin absorption in bovine neonates occurs via receptor-mediated and nonspecific transcytosis (Staley and Bush, 1985; Baumrucker et al., 2010), which may become saturated as increasing quantities of IgG are present at the apical surface. This results in reduced AEA at higher IgG intakes (Quigley et al., 2002; Lopez et al., 2022; Mitman et al., 2025). Importantly, in this study reduced calculated AEA did not translate into poorer passive immunity, as calves receiving greater IgG mass still achieved higher serum IgG concentrations (Osaka et al., 2014; Saldana et al., 2019; Davies et al., 2025).

The time of birth was also associated with AEA, with calves born during the morning or the afternoon or evening having higher AEA than those born overnight. One possible explanation for the association between time of birth and AEA is variation in the interval from birth to first colostrum feeding resulting from differences in staffing during daytime and overnight periods. However, because feeding delay was not recorded, this mechanism could not be evaluated directly in the present study. Delaying colostrum feeding has been consistently associated with reduced IgG absorption efficiency (Stott et al., 1979; Besser et al., 1988; Fischer et al., 2018), with declines observed even when feeding is delayed by as little as 30 min (Rajala and Castrén, 1995). This reduction reflects the rapid decline in intestinal permeability to immunoglobulins during the first 24 h of life as gut closure progresses (Stott et al., 1979; Bush and Staley, 1980). Adequate staffing during peak calving periods may therefore improve AEA by minimizing delays to first colostrum feeding in seasonal block-calving dairy systems.

The higher estimated AEA among calves born in May was based on only 5 calves. This exploratory estimate is therefore highly uncertain and requires confirmation in a larger, more evenly distributed cohort.

Sex, birth weight, and calving score were not associated with AEA. The lack of association with calving score must also be interpreted with caution, as most calvings proceeded unassisted (unassisted = 193 out of 213, easy pull = 16 out of 213, hard pull = 4 out of 213, surgery = 0), which is typical of pasture-based block-calving systems. Studies in Ireland report dystocia rates of approximately 5.8% to 6.8% (Mee et al., 2011), whereas higher rates have been reported in more intensive indoor systems (e.g., 13.7%; Tsaousioti et al., 2023). Dystocia may influence AEA indirectly through its effects on calf vitality (Murray et al., 2015) and acid–base status (Staley and Bush, 1985), rather than acting as a direct determinant of IgG absorption.

No clear evidence of a breed association was detected under the feeding protocol in this study. This suggests that, within this cohort, beef-cross calves did not differ detectably from dairy-bred calves in AEA when colostrum management was broadly similar. Evidence from related work in beef calves indicates that colostrum replacement strategy and IgG delivery can influence IgG absorption efficiency (Mitman et al., 2025), supporting the interpretation that management-related factors may be more important than breed alone. This finding is noteworthy given the rapid expansion of beef-on-dairy breeding strategies globally (Berry, 2021).

Several limitations should be considered when interpreting the findings of this study. First, AEA is a calculated outcome derived from serum IgG concentration, estimated plasma volume (based on body weight), and total IgG intake. Consequently, total IgG intake and body weight are not fully independent of the outcome, creating the potential for mathematical coupling when evaluated as explanatory variables. A sensitivity analysis excluding total IgG intake from the model demonstrated that the estimated effects of month of birth and feeding time were essentially unchanged, indicating that these associations were robust to exclusion of this variable. The AEA calculation also estimates plasma volume as 0.09 × body weight rather than measuring it directly. Although this approach is widely used, plasma volume may vary among neonatal calves because of differences in hydration status and physiological adaptation after birth. Consequently, variation in true plasma volume may have introduced some error into the estimated AEA values.

Second, calves were not sampled immediately after birth to establish baseline circulating immunoglobulin concentrations before colostrum intake. Although previous evidence shows that colostrum-deprived calves typically possess only minimal immunoglobulin concentrations during the initial 3 d postpartum (Clover and Zarkower, 1980), the absence of pre-colostrum samples prevents complete exclusion of unobserved suckling or trans-placental transfer of antibodies. Calves that suckled without being observed may have consumed additional colostrum, resulting in underestimation of total IgG intake and overestimation of AEA. Therefore, the reported AEA values should be interpreted with this possibility in mind.

Finally, several management variables could not be fully accounted for. The distribution of births was uneven across months, with relatively few calves born in May, limiting the precision of monthly comparisons. Although total IgG consumed was quantified, microbial load was not measured. Given that bacterial contamination can reduce IgG absorption efficiency (James et al., 1981; Gelsinger et al., 2015), this may have contributed to unexplained variation in AEA. The number of CR sachets and subsequent pooled colostrum or transition milk feedings reflected routine farm management rather than a pre-specified allocation protocol. Differences in administration of 1 versus 2 sachets were attributable to variation in staff feeding practices rather than a standardized decision-making process. Although the decision was not based on recorded calf characteristics, the potential for unmeasured confounding cannot be completely excluded. The exact interval from birth to first colostrum feeding was not recorded, so time of birth served as a proxy for potential differences in feeding delay. Likewise, although all calves were sampled at least 6 h after the final colostrum or transition milk feeding, the age at blood sampling (24–36 h) and the interval between the final feeding and blood collection were not standardized and may have varied among calves. These intervals may also have differed systematically among time of birth categories because of routine feeding and sampling schedules. Consequently, the observed association between time of birth and calculated AEA could partly reflect differences in feeding or sampling timing rather than time of birth itself. These variables were not available for analysis and therefore could not be evaluated further. In addition, calves could share exposure to one or more pooled maternal colostrum or transition milk batches. Because individual calves received milk from multiple sequential batches during the first 24 h, calves could not be assigned to a single batch for assessment of clustering. Despite these limitations, the relatively large cohort and detailed recording of colostrum feeding and IgG intake strengthen confidence in the observed associations.

This study demonstrates that, in this pasture-based spring block-calving herd, calculated AEA was primarily associated with colostrum management factors, particularly the timing of calving and total IgG intake. The association with total IgG intake must be interpreted cautiously because intake is incorporated into the calculation of AEA. Despite differences in production system, these determinants appear consistent with those reported in housed dairy herds. The absence of an association with breed suggests that beef-cross calves do not differ from dairy-bred calves in their capacity to absorb IgG when managed under similar conditions.
